# Therapeutic Effect of Endothelin-Converting Enzyme Inhibitor on Chronic Kidney Disease through the Inhibition of Endoplasmic Reticulum Stress and the NLRP3 Inflammasome

**DOI:** 10.3390/biomedicines9040398

**Published:** 2021-04-07

**Authors:** Yung-Ho Hsu, Cai-Mei Zheng, Chu-Lin Chou, Yi-Jie Chen, Yu-Hsuan Lee, Yuh-Feng Lin, Hui-Wen Chiu

**Affiliations:** 1 Department of Internal Medicine, School of Medicine, College of Medicine, Taipei Medical University, Taipei 11031, Taiwan; yhhsu@s.tmu.edu.tw (Y.-H.H.); 11044@s.tmu.edu.tw (C.-M.Z.); chulin.chou@tmu.edu.tw (C.-L.C.); 2 TMU Research Center of Urology and Kidney, Taipei Medical University, Taipei 11031, Taiwan; 3 Division of Nephrology, Department of Internal Medicine, Hsin Kuo Min Hospital, Taipei Medical University, Taoyuan City 320001, Taiwan; 4 Division of Nephrology, Department of Internal Medicine, Shuang Ho Hospital, Taipei Medical University, New Taipei City 23561, Taiwan; 5 Graduate Institute of Clinical Medicine, College of Medicine, Taipei Medical University, Taipei 11031, Taiwan; chenyj1108@tmu.edu.tw; 6 Department of Cosmeceutics, China Medical University, Taichung 406040, Taiwan; yhlee@mail.cmu.edu.tw; 7 Department of Internal Medicine, School of Medicine, National Defense Medical Center, Taipei 11490, Taiwan; 8 Department of Medical Research, Shuang Ho Hospital, Taipei Medical University, New Taipei City 23561, Taiwan

**Keywords:** chronic kidney disease, endothelin-converting enzymes, endoplasmic reticulum stress, autophagy, inflammasome

## Abstract

Chronic inflammation and oxidative stress significantly contribute to the development and progression of chronic kidney disease (CKD). The NOD-like receptor family pyrin containing domain-3 (NLRP3) inflammasome plays a key role in the inflammatory response. The renal endothelin (ET) system is activated in all cases of CKD. Furthermore, ET-1 promotes renal cellular injury, inflammation, fibrosis and proteinuria. Endothelin-converting enzymes (ECEs) facilitate the final processing step of ET synthesis. However, the roles of ECEs in CKD are not clear. In this study, we investigated the effects of ETs and ECEs on kidney cells. We found that ET-1 and ET-2 expression was significantly upregulated in the renal tissues of CKD patients. ET-1 and ET-2 showed no cytotoxicity on human kidney tubular epithelial cells. However, ET-1 and ET-2 caused endoplasmic reticulum (ER) stress and NLRP3 inflammasome activation in tubular epithelial cells. The ECE inhibitor phosphoramidon induced autophagy. Furthermore, phosphoramidon inhibited ER stress and the NLRP3 inflammasome in tubular epithelial cells. In an adenine diet-induced CKD mouse model, phosphoramidon attenuated the progression of CKD by regulating autophagy, the NLRP3 inflammasome and ER stress. In summary, these findings showed a new strategy to delay CKD progression by inhibiting ECEs through autophagy activation and restraining ER stress and the NLRP3 inflammasome.

## 1. Introduction

There is a steady increase in the number of patients worldwide with chronic kidney disease (CKD), and diabetes and hypertension are the major causes [1,2]. Ongoing obesity/diabetes is widespread and affects older individuals worldwide, and current therapies only partially slow the progression of CKD; thus, there is an urgent need for other effective therapeutic agents [1,3,4]. Although many potential drug targets are being developed, the endothelin (ET) system has received particularly high attention. Previous studies have demonstrated that the renal ET system is activated in all cases of CKD [5]. There are three 21-amino-acid peptides (ET-1, ET-2, and ET-3) in the ET family. ET-1 has been the most widely studied ET in human diseases [5]. ET-1 regulates sodium homeostasis and systemic blood pressure [6]. ET precursors are processed by 2 proteases into the active forms. Preproendothelins are cleaved by furin-like endopeptidase to create inactive intermediates, which are termed proendothelins or big ETs [7]. This processing is mediated by a family of membrane-bound zinc metalloproteases, termed endothelin-converting enzymes (ECEs). These proteases facilitate to the final processing step [8]. ETs bind to two types of receptors, endothelin A receptor (ET_A_) and the endothelin B receptor (ET_B_) [9]. ET_A_ and ET_B_ are located on vascular smooth muscle cells, and their activation induces vasoconstriction. ET_B_ is primarily present on vascular endothelial cells, where activation causes vasodilation through prostaglandin and nitric oxide release [10,11]. Within the kidney, ET-1 is produced by mesangial cells, tubular epithelial cells, podocytes and the renal collecting duct. Previously, it was reported that ET-1 binding to ET_A_ promoted renal cellular injury, inflammation, fibrosis and proteinuria [9]. Plasma ET-1 correlates with the degree of albuminuria and the severity of CKD [12,13]. The recently published results (SONAR study) indicated that a selective ET_A_ antagonist atrasentan improved renal outcomes in patients with diabetes and CKD [14]. It has been reported that the prepro-ET-1-ECE-1-ET-1-ET_A_ pathway is responsible for cell growth, vasoconstriction and inflammation, among other processes [7]. It is often presumed that ET-2 could mimic the actions of the more plentiful ET-1 and current pharmacological interventions to inhibit the ET system could also restrain the effects of ET-2. These hypotheses have focused on ET-1. However, ET-2 has been less well studied than ET-1 [15]. Previous research has shown that treatment with SLV338, a neutral endopeptidase (NEP)/ECE inhibitor, diminished renal tissue damage (glomerulosclerosis, interstitial fibrosis and renal arterial remodeling) but did not significantly affect blood pressure [16]. Phosphoramidon, which is an ECE inhibitor, inhibited lipopolysaccharide-induced acute lung injury [17]. However, the complete mechanism by which ECE inhibitors affect cellular signaling in CKD is unknown and needs further investigation.

Recent evidence shows that overactivation of the ET-1 system may induce the endoplasmic reticulum (ER) stress response in pulmonary aortic smooth muscle cells and placental tissue [18,19]. ER stress is a type of cellular stress that induces the accumulation of unfolded proteins through ER dysfunction and may cause cell damage. When ER stress is activated, cells induce the adaptive unfolded protein response (UPR) to maintain homeostasis. The UPR pathway is regulated by three major pathways: pancreatic eukaryotic translation initiation factor 2α (eIF2α) kinase (PERK), activating transcription factor 6 (ATF6) and inositol-requiring protein 1 (IRE1) [20,21]. Many studies have demonstrated a relationship between the UPR pathway and tubular and glomerular cell damage in various kidney diseases [22]. Previous research has shown that ET-1 induces renal immune activation and/or inflammation by promoting renal ER stress through ET_A_ [23]. Furthermore, ER stress triggers NOD-like receptor family, pyrin containing domain-3 (NLRP3) inflammasome activation, which promotes the inflammatory response, in the renal tubule [24]. NLRP3 inflammasome activation plays a key role in kidney injury and has been identified as a therapeutic target in the treatment of CKD patients [25]. Previous studies have demonstrated that autophagy can inhibit the NLRP3 inflammasome [26,27]. Autophagy is a metabolic pathway that maintains the dynamic balance of cells [28]. Autophagy is involved in human kidney diseases such as acute kidney injury, CKD, polycystic kidney diseases and diabetic nephropathies [29]. The aim of this study was to investigate whether ETs activate the NLRP3 inflammasome and ER stress in kidney cells. Furthermore, we evaluated whether phosphoramidon, which is an ECE inhibitor, ameliorates the progression of CKD regulating autophagy, the NLRP3 inflammasome and ER stress.

## 2. Materials and Methods

### 2.1. Microarray Analysis

Raw CKD patient data from the microarray dataset GSE66494 were obtained from the Gene Expression Omnibus (GEO) database (https://www.ncbi.nlm.nih.gov/geo/; accessed on 1 August 2017). These data were normalized with GeneSpring v11 software (Agilent Technologies, Santa Clara, CA, USA) as log_2_ values. The boxplot, which was produced using SPSS 22 software (IBM, Armonk, NY, USA), showed the transcriptional activity between CKD patients and healthy individuals.

### 2.2. Cell Culture and Drug Treatment

The human kidney proximal tubular epithelial cell line HK-2 was acquired from the American Type Culture Collection (CRL2190) and approved according to the guidelines of our institute (Shuang Ho Hospital, Taipei Medical University, Taiwan; SHH-0186; 24 December 2019). Cells were cultured in keratinocyte serum-free (KCSF) medium with bovine pituitary extract (40 µg/mL) and recombinant epidermal growth factor (5 ng/mL) (Gibco BRL, Grand Island, NY, USA) at 37 °C and 5% CO_2_. Cells were used between the 20th and 30th passages. Fresh solutions of ET-1, ET-2, Thapsigargin (Cayman Chemical, Ann Arbor, MI, USA), lipopolysaccharide (LPS) (Sigma-Aldrich Corp., St. Louis, MO, USA), chloroquine (Sigma-Aldrich Corp.) and phosphoramidon (ApexBio, Boston, MA, USA) were prepared before each experiment. These reagents were added to the culture medium and mixed gently.

### 2.3. Cell Viability Assay

Cell viability was evaluated using a sulforhodamine B (SRB) assay. Cells were incubated in 96-well plates. Then, the cells were fixed with a trichloroacetic acid solution for 1 h. The supernatant was removed, and the plates were washed five times and air dried. SRB (Sigma-Aldrich Corp.) was added to each of the wells for 1 h. After staining, the residual dye was removed, and the wells were washed five times with 1% acetic acid. Tris buffer (20 mM) was added, and then the absorbance of the solution was measured on an absorbance microplate reader (Molecular Devices, Sunnyvale, CA, USA) at a wavelength of 562 nm. The absorbance of untreated cells was used as the reference to calculate 100% cell viability.

### 2.4. Western Blotting

Cells were harvested after treatment and lysed with RIPA lysis buffer (Genestar, Taipei, Taiwan). Equal amounts of protein (30 µg/lane) were loaded and separated on a sodium dodecyl sulfate (SDS) gel. The gel was subjected to electrophoresis, blotted, and probed using primary and secondary antibodies, and the targets were analyzed using a chemiluminescence detection system (Thermo Fisher Scientific, Waltham, MA, USA). Anti-IRE1α (3294), anti-eIF2α (5324), anti-p-eIF2α (3398), anti-LC3 (4108) and anti-Beclin 1 (3738) antibodies were obtained from Cell Signaling Technology (Ipswich, MA, USA); anti-ATF6 (24169-1-AP), anti-Caspase 1 (22915-1-AP) and anti-GAPDH (60004-1-1g) antibodies were obtained from Proteintech Group (Chicago, IL, USA); anti-ET-1 (A0686) antibody was obtained from ABclonal Inc. (Boston, MA, USA); anti-ET-2 (BS-11280R) antibody was obtained from Bioss antibodies Inc. (Woburn, MA, USA), anti-p62 (PM045) antibody was obtained from MBL (Nagoya, Japan); the anti-NLRP3 (ab214185) antibody was obtained from Abcam (Cambridge, MA, USA); and the anti-ASC (AG-25B-0006) antibody was obtained from Adipogen (San Diego, CA, USA).

### 2.5. Immunofluorescence Assay

The cells were seeded on cover glass. After phosphoramidon treatment, the cells were fixed with methanol or 4% paraformaldehyde. The cells were blocked with 1% bovine serum albumin for 30 min and probed with an anti-LC3 antibody (MBL, Nagoya, Japan) for 1 h. After being washed, the cells were incubated with goat anti-rabbit DyLight™ 488 (Jackson ImmunoResearch Laboratories, West Grove, PA, USA) antibodies for 1 h and then stained with 4’,6-diamidino-2-phenylindole (DAPI) (Invitrogen). Images were obtained with a fluorescence microscope or confocal microscope (Leica TCS SP5, Mannheim, Germany).

### 2.6. Mouse Model of Adenine Diet-Induced CKD

Then, 8-week-old male C57BL/6 mice were obtained from the National Laboratory Animal Center (Taiwan). All protocols were conducted and approved according to the guidelines of our institute (Institutional Animal Care and Use Committee of Taipei Medical University, Taiwan; approval number: LAC-2019-0521; 1 August 2020). The mouse model of adenine diet-induced CKD is based on previous publications [26,30]. The mice were divided into the following four groups (five animals/group): chow-fed mice (Normal group), adenine-fed and saline-injected mice (CKD group), adenine-fed and low-concentration (5 mg/kg) phosphoramidon-injected mice (CKD+L group) and adenine-fed and high-concentration (10 mg/kg) phosphoramidon-injected mice (CKD+H group). In the CKD group, the mice were fed a 0.2% adenine diet for 5 weeks to induce CKD. In the CKD+L and CKD+H groups, the mice were fed a 0.2% adenine-containing diet for 1 week and then intraperitoneally (i.p.) injected with phosphoramidon two times per week for 4 weeks while being fed the 0.2% adenine diet. The mice were sacrificed by CO_2_ exposure. 

### 2.7. Biochemical Tests

Whole blood samples were collected by intracardiac puncture. Furthermore, blood samples were centrifuged at 2000× *g* for 20 min to separate the serum. Creatinine and blood urea nitrogen (BUN) were analyzed.

### 2.8. Histopathological and Immunohistochemical Staining

The kidney tissue sections were fixed with formalin and then embedded in paraffin. The kidney sections were dewaxed and rehydrated. After being blocked in hydrogen peroxide (3%) for 20 min, the sections were subjected to antigen retrieval. Then, the tissue sections were stained with hematoxylin and eosin (H&E) to evaluate histopathological changes. For immunohistochemical staining, the dewaxed sections were blocked in 3% hydrogen peroxide and incubated with anti-IRE1α (Novus Biologicals, Littleton, CO, USA), anti-LC3 (MBL, Nagoya, Japan), anti-ET-1 (ABclonal Inc., Boston, MA, USA), anti-ET-2 (Bioss antibodies Inc., Woburn, MA, USA) or anti-NLRP3 (Abcam, Cambridge, MA, USA) antibodies at room temperature for 2 h. Then, the slides were incubated with a secondary antibody at room temperature for 1 h, and a STARR TREK Universal HRP detection kit (Biocare Medical, Concord, CA, USA) was used. Finally, the slides were stained with hematoxylin and observed using a light microscope. The images were quantified the positive cells by ImageJ plugins. The IHC of positive percentage areas were analyzed in 10 fields of view.

### 2.9. Masson Staining

Masson trichrome staining was analyzed according to the protocol (ScyTek Lab., Logan, UT, USA).

### 2.10. Statistical Analysis

The data are shown as the means ± standard deviation (SD), and the differences between groups were assessed using a two-sample *t*-test or one-way analysis of variance with a post hoc Dunnett’s multiple comparison test. In all statistical tests, *p* < 0.05 was considered statistically significant.

## 3. Results

### 3.1. ET Expression in CKD Patients and ET-Induced ER Stress and NLRP3 Inflammasome Activation in Human Kidney Cells

**Figure 1:**
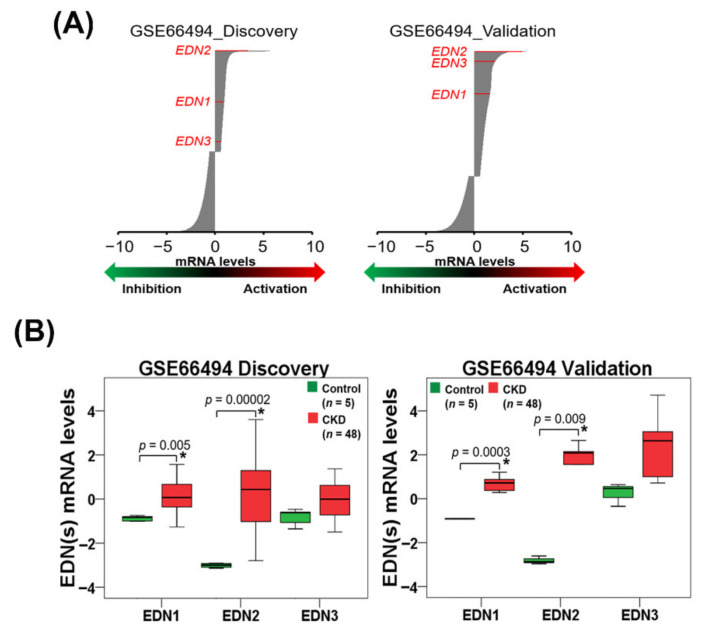
*EDN1*, *EDN2* and *EDN3* expression in renal tissues of healthy individuals and chronic kidney disease (CKD) patients. (**A**) The *EDN1*, *EDN2* and *EDN3* mRNA levels in the renal tissues of CKD patients (discovery and validation cohort in GSE66494) at a 1.5-fold change (FC) threshold. (**B**) The mRNA levels of *EDN1*, *EDN2* and *EDN3* were upregulated in the renal tissues of CKD patients (discovery and validation cohort in GSE66494). * *p* < 0.05 compared with the control.

**Figure 2:**
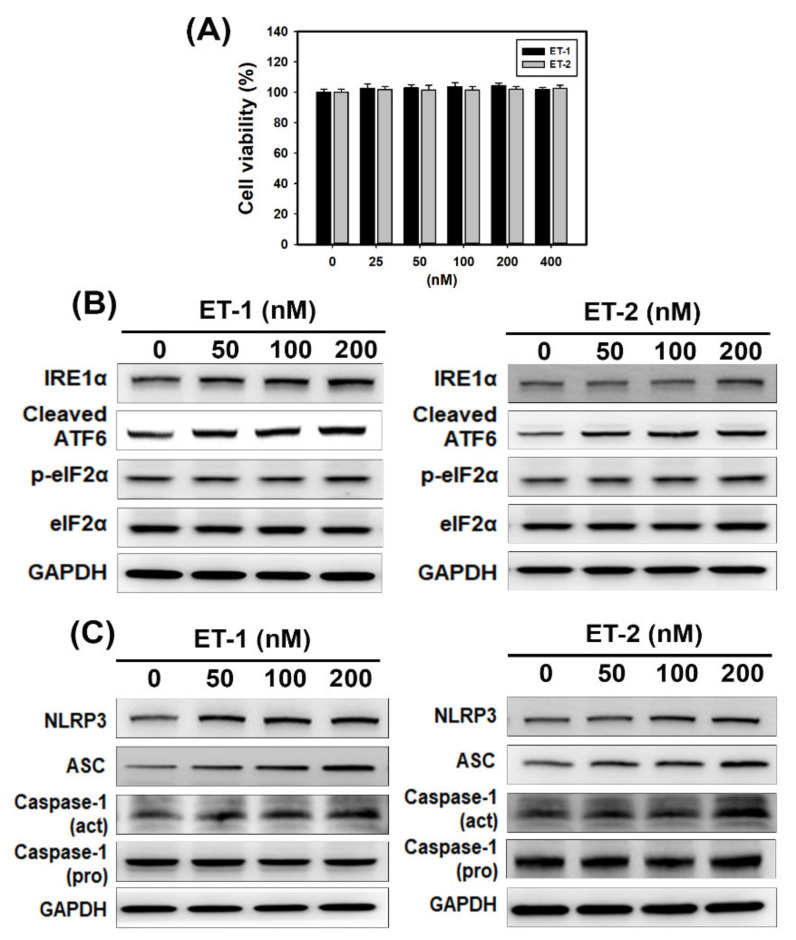
Cell viability, endoplasmic reticulum (ER) stress and the NLRP3 inflammasome in HK-2 cells treated with ET-1 or ET-2. (**A**) Cell viability of ET-1- or ET-2-treated HK-2 cells. Data were presented as the means ± standard deviation of three independent experiments. (**B**) Western blot analysis of ER stress-associated protein expression in HK-2 cells. (**C**) Western blot analysis of NLRP3 inflammasome-associated protein expression in HK-2 cells. Cells were treated with various concentrations of ET-1 or ET-2 for 24 h.

We first analyzed the transcriptional profiles of *EDN1* (ET-1), *EDN2* (ET-2) and *EDN3* (ET-3) in kidney tissues from CKD patients in the GEO database (Figure 1A). The data showed that the mRNA levels of *EDN1* and *EDN2* but not *EDN3* were significantly (*p* < 0.05) upregulated in kidney tissues from CKD patients compared to healthy individuals (Figure 1B). Next, we investigated whether ET-1 and ET-2 induce ER stress in HK-2 human kidney proximal tubular epithelial cells. After treatment with ET-1 or ET-2 for 24 h, HK-2 cell viability was not changed, as evidenced by SRB assays (Figure 2A). Therefore, ET-1 or ET-2 showed no cytotoxicity on human kidney proximal tubular epithelial cells. Furthermore, we found that the expression levels of UPR-related proteins, including IRE1α and cleaved ATF6, increased in HK-2 cells treated with ET-1 or ET-2 (Figure 2B and Figure S1). However, there is no significant difference on the expression of phosphorylated eIF2α in HK-2 cells treated with ET-1 or ET-2 (Figure 2B and Figure S1). We evaluated whether ET-1 or ET-2 triggers NLRP3 inflammasome activation. As shown in Figure 2C and Figure S2, ET-1 and ET-2 treatment increased NLRP3, ASC and cleaved caspase-1 expression in HK-2 cells. These findings indicate that ET-2 and ET-2 induce ER stress and the NLRP3 inflammasome in human kidney cells.

### 3.2. The ECE Inhibitor Phosphoramidon Triggers Autophagy in Human Kidney Cells

**Figure 3:**
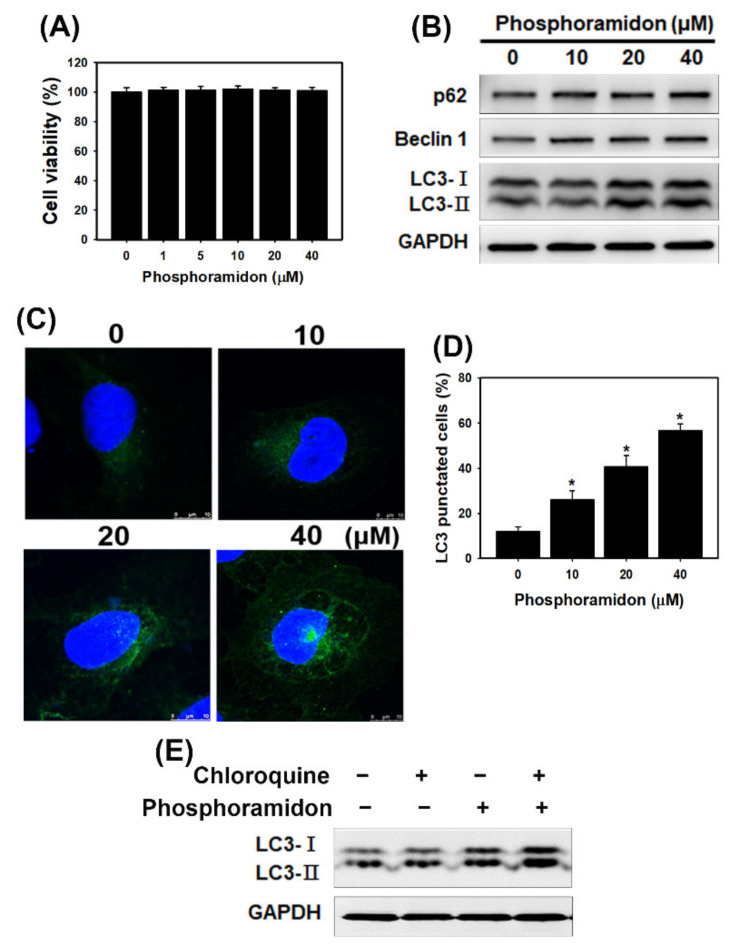
Phosphoramidon triggers autophagy in HK-2 cells. (**A**) Cell viability of phosphoramidon-treated cells. Cells were treated with several concentrations of phosphoramidon for 24 h. Data were presented as the means ± standard deviation of three independent experiments. (**B**) The protein levels of p62, beclin 1 and LC3 in HK-2 cells treated with phosphoramidon for 24 h. (**C**) Imaging of LC3 by confocal immunofluorescence microscopy following 24 h of treatment with phosphoramidon. Scale bar = 10 µm. (**D**) Quantification of punctate LC3 staining. Cells were treated with various concentrations of phosphoramidon for 24 h. * *p* < 0.05 compared with the control. Data were presented as the means ± standard deviation of three independent experiments. (**E**) Western blotting of LC3-I and LC3-II expression in HK-2 cells. The cells were pretreated with chloroquine (5 µM) for 1 h and then treated with phosphoramidon (20 µM) for 24 h.

To determine whether the ECE inhibitor phosphoramidon affects HK-2 cell viability, the cells were treated with phosphoramidon at the indicated concentrations (Figure 3A). The results showed that phosphoramidon did not cause significant changes in cell viability. There have been few articles describing the relationship between ECE inhibitors and autophagy. Therefore, we examined whether phosphoramidon induced autophagy. We analyzed autophagy-related proteins by Western blotting (Figure 3B). The levels of beclin 1, LC3-II and p62 were increased in cells treated with phosphoramidon (Figure S3). In addition, we analyzed the percentage of cells with punctate LC3 staining by fluorescence microscopy (Figure 3C,D). The results indicated that treatment with phosphoramidon increased LC3 puncta in a concentration-dependent manner in HK-2 cells. Increases in autophagic markers may represent raised generation of autophagosomes in autophagic flux and/or inhibition of autophagosomal maturation and degradation. Chloroquine, which inhibits lysosomal clearance of autophagosomes, are due to produced autophagic flux. The results found that the LC3-II expression was increased by phosphoramidon treatment with chloroquine (Figure 3E and Figure S3). Therefore, phosphoramidon treatment caused autophagic flux in HK-2 cells. These results suggest that the ECE inhibitor phosphoramidon triggers autophagy in human kidney cells.

### 3.3. Phosphoramidon Alleviates ER Stress and NLRP3 Inflammasome Activation in Human Kidney Cells

**Figure 4:**
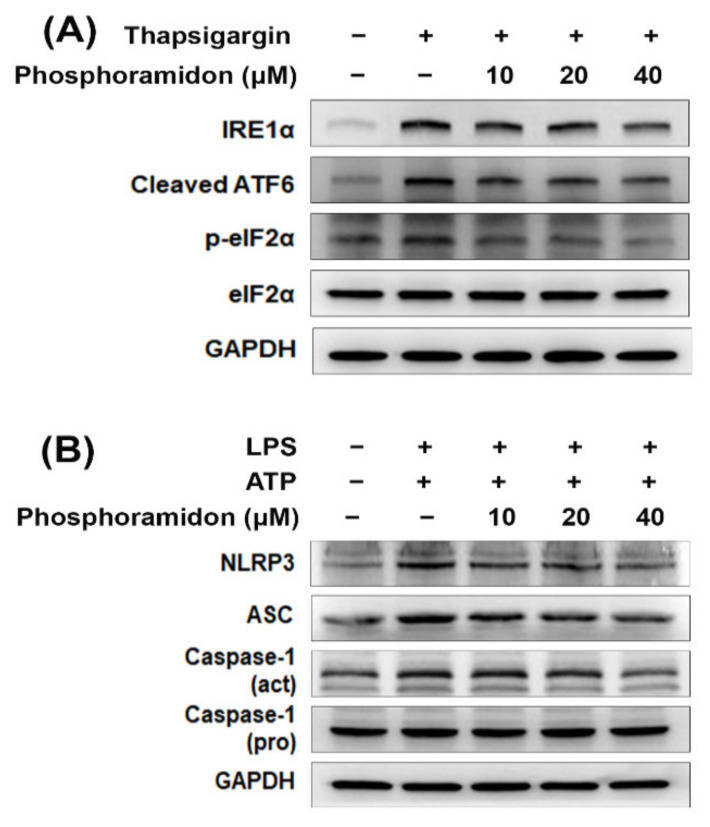
Phosphoramidon reduces ER stress and the NLRP3 inflammasome in HK-2 cells. (**A**) Western blot analysis of ER stress-related protein expression in HK-2 cells. Cells were pretreated with phosphoramidon for 6 h and then treated with 150 nM thapsigargin for 18 h. (**B**) Western blot analysis of NLRP3 inflammasome-related protein expression in HK-2 cells. Cells were incubated for 24 h with phosphoramidon and 1 µg/mL LPS and then treated with 2 mM ATP for 2 h.

To address whether phosphoramidon inhibits ER stress, the ER stress activator thapsigargin was used. The results showed that thapsigargin increased the expression of IRE1α and cleaved ATF6 but did not increase phosphorylated eIF2α (Figure 4A and Figure S4). Phosphoramidon inhibited the thapsigargin-induced expression of UPR-related proteins. Furthermore, we assessed whether phosphoramidon restrains NLRP3 inflammasome activation. HK-2 cells were treated with LPS plus ATP, which increased NLRP3, ASC and cleaved caspase-1 expression (Figure 4B and Figure S5). Phosphoramidon suppressed the LPS- and ATP-induced activation of the NLRP3 inflammasome. Taken together, these results showed that phosphoramidon inhibited ER stress and the NLRP3 inflammasome in kidney cells.

### 3.4. Phosphoramidon Attenuates the Progression of CKD by Regulating Autophagy, the NLRP3 Inflammasome and ER Stress in a Mouse Model

**Figure 5:**
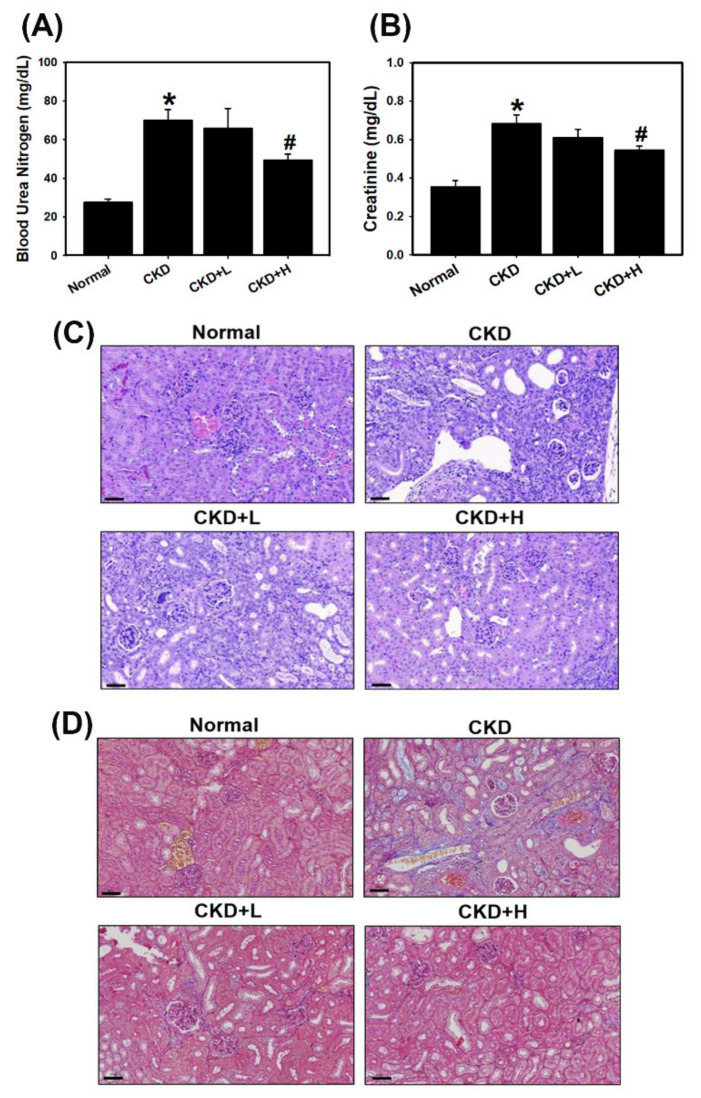
Biochemical and histopathological analyses of a mouse model of adenine diet-induced CKD. Renal function was evaluated by analyzing blood urea nitrogen (BUN) (**A**) and creatinine (**B**) levels (five mice per group). * *p* < 0.05 compared with the normal group. # *p* < 0.05 compared with the CKD group. Data are presented as the means ± standard deviation. Representative micrographs of H&E (**C**) and Masson’s trichrome (**D**) staining in the indicated groups in the adenine diet-induced CKD model. Scale bar = 60 µm.

**Figure 6:**
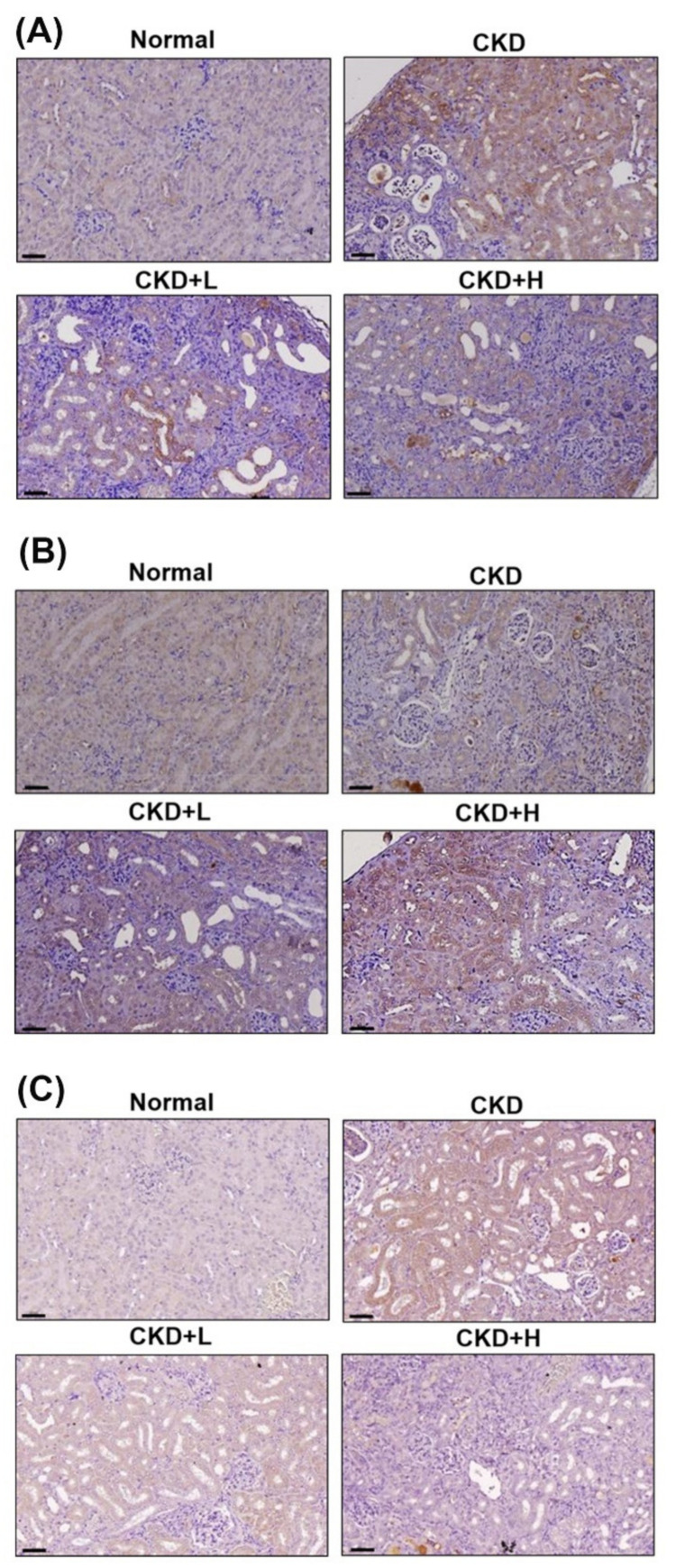
Phosphoramidon regulates ER stress, autophagy and the NLRP3 inflammasome in the adenine diet-induced CKD model. The protein expression of IRE1α (**A**), LC3 (**B**) and NLRP3 (**C**) in kidney sections in the indicated groups. Scale bar = 60 µm.

In a mouse model of adenine diet-induced CKD, mice were observed for 5 weeks following treatment with phosphoramidon, after which their body weights were determined and biochemical examinations were performed. The CKD, low-concentration (CKD+L) and high-concentration (CKD+H) phosphoramidon group had lower body weights than the control group (Figure S6). As shown in Figure 5A,B, serum creatinine and BUN concentrations were elevated after adenine feeding. CKD+H inhibited adenine-induced creatinine and BUN levels. Moreover, histopathological evaluation of the kidney tissues in the CKD group showed tubular dilation, glomerular atrophy, loss of the brush border and interstitial inflammatory cell infiltration (Figure 5C). Treatment with low-concentration (5 mg/kg) and high-concentration (10 mg/kg) phosphoramidon greatly ameliorated these abnormalities. The evaluation of renal fibrosis was used by Masson’s trichrome staining (Figure 5D). We found that fibrosis was constrained in phosphoramidon-treated mice (CKD+L and CKD+H) in comparison to that in adenine-treated mice (CKD group) (Table S1). In addition, we examined the effects of phosphoramidon on ETs, ER stress, autophagy and the NLRP3 inflammasome in kidney tissue sections. The results showed that ET-1, ET-2, IRE1α, LC3 and NLRP3 expression was increased in the CKD group (Figure 6, Figure S7, Figure S8 and Table S1). The CKD+L and CKD+H groups exhibited inhibited expression of ET-2 and NLRP3 but increased LC3 expression compared with that in the CKD group (Table S1). The CKD+H groups suppressed the ET-1 and IRE1α expression (Table S1). Moreover, kidney tissues were analyzed by Western blotting and the results were similar to immunohistochemical staining (Figure S9). These results confirm that phosphoramidon ameliorates kidney function through inhibiting ER stress and the NLRP3 inflammasome and inducing autophagy in kidney cells.

## 4. Discussion

ER stress is a type of cellular stress that results from the accumulation of unfolded proteins in the ER [31]. Recent evidence has shown that ER stress is involved in renal apoptosis and injury [32]. Evidence has demonstrated that both ET-1 and ER stress are upregulated in many renal diseases [33,34,35]. Therefore, overactivation of the ET-1 system may cause the renal ER stress response [20]. The results of the present study indicate that the mRNA levels of EDN1 (ET-1) and EDN2 (ET-2) were significantly increased in kidney tissues in CKD patients compared to healthy individuals (Figure 1B). Previously, it was reported that treatment with a neutral endopeptidase (NEP)/ECE inhibitor diminished renal tissue damage [16]. In this study, phosphoramidon inhibited adenine-induced creatinine and BUN levels (Figure 5A,B). Moreover, phosphoramidon greatly ameliorated histopathological lesions and fibrosis in the kidney (Figure 5C,D and Table S1). In addition, we found that the expression levels of UPR-related proteins increased with ET-1 or ET-2 treatment in HK-2 cells (Figure 2B and Figure S2). Therefore, ET-1 and ET-2 can induce ER stress in human kidney cells. Our results also revealed that phosphoramidon alleviated ER stress (Figure 4A and Figure S4). In an in vivo experiment, phosphoramidon ameliorated adenine-induced ET-1, ET-2 and ER stress (Figure 6A, Figure S7, Figure S9 and Table S1).

Inflammasomes are multiprotein innate immune complexes. The NLRP3 inflammasome has been shown to contribute to many acute and chronic kidney diseases via canonical and noncanonical pathways that modulate apoptosis, inflammation, fibrosis and pyroptosis [36]. Our previous study showed that NLRP3 expression levels were increased in renal tissues or peripheral blood monocytic cells (PBMCs) from CKD patients compared with those of healthy donors [26]. However, little information is available about the relationship between ETs and inflammasomes. Recent evidence shows that diabetes-mediated increases in ET-1 in hippocampal neurons induce NLRP3 activation and inflammation [37]. In the present study, ET-1 and ET-2 triggered NLRP3 inflammasome activation in human kidney cells (Figure 2C and Figure S2). Phosphoramidon suppressed the LPS- and ATP-induced activation of the NLRP3 inflammasome (Figure 4B and Figure S5). Furthermore, phosphoramidon inhibited NLRP3 expression in kidney tissue sections in a mouse model of adenine diet-induced CKD (Figure 6C, Figure S8 and Table S1).

The present study demonstrated that phosphoramidon-mediated inhibition of ETs increased LC3 puncta and autophagy-associated proteins in human kidney cells (Figure 3 and Figure S3). CKD mice treated with phosphoramidon exhibited increased LC3 expression in kidney tissue sections compared with that in mice in the CKD group (Figure 6B, Figure S8 and Table S1). Therefore, phosphoramidon triggers autophagy in kidney cells. Furthermore, phosphoramidon did not cause significant changes in cell viability (Figure 3A). Accumulating evidence has revealed that autophagy plays a key role in kidney health, disease and aging. The renoprotective functions of autophagy in podocytes and epithelial renal cells are regulated by the clearance of altered mitochondria, the removal of protein aggregates, and the inhibition of inflammation and cell death [38]. Previous research has shown that autophagy attenuates tubulointerstitial fibrosis by suppressing the NLRP3 inflammasome and transforming growth factor-β [39]. Furthermore, autophagy has cytoprotective effects that maintain glomerular homeostasis under physiological and pathological conditions. The interruption of autophagic flux in podocytes induces cytoplasmic accumulation of damaged organelles and protein aggregates, causing ER stress, oxidative stress, inflammation and apoptosis [40]. Another recent study indicated that ET-1 decreased autophagy and that the ET_A_ receptor antagonist BQ123 increased autophagy in H9C2 myoblasts. Moreover, cardiac ET_A_ receptor deletion rescues aging-associated cardiac hypertrophy and contractile dysfunction via autophagy induction [41]. Pacheco-Quinto and Eckman found that ECEs degraded intracellular β-amyloid, which is a possible cause of the neuronal toxicity that is typical of Alzheimer’s disease, through autophagy regulation [42]. However, there have been few published studies describing the relationship between ECEs and autophagy in kidney cells. In this report, we demonstrate that the ECE inhibitor phosphoramidon induces autophagy without any injury or stress in kidney cells. These findings support further evaluation of phosphoramidon or other ECE inhibitors as potential treatments for human inflammatory disease.

## 5. Conclusions

**Figure 7:**
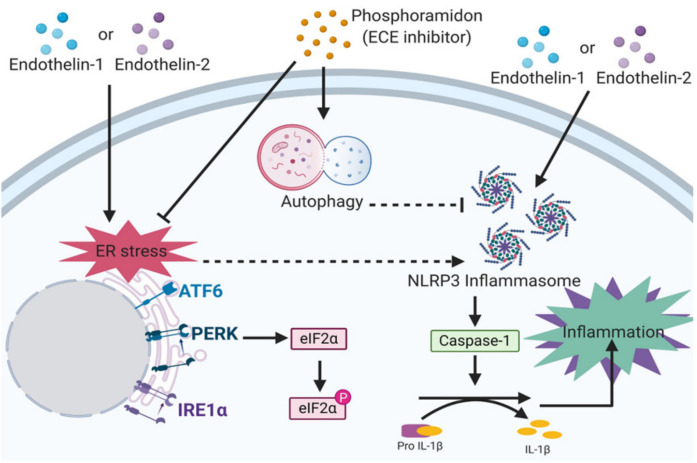
Phosphoramidon exerts a renoprotective effect on CKD progression. ET-1 and ET-2 induce ER stress and the NLRP3 inflammasome, which cause inflammation in kidney cells. The endothelin-converting enzymes (ECE) inhibitor phosphoramidon triggers autophagy. Furthermore, phosphoramidon inhibits ER stress and the NLRP3 inflammasome in kidney cells. Therefore, phosphoramidon is an effective therapeutic treatment for CKD progression. The point arrows indicated activation or induction. The blunt ends indicated inhibition. The solid lines indicated direct interaction. The dashed lines indicated indirect interaction. The figure was created with BioRender.com.

Our present study provides evidence that ET-1 and ET-2 expression was significantly upregulated in CKD patients. ETs induced ER stress and the NLRP3 inflammasome in human kidney cells. The ECE inhibitor phosphoramidon triggered autophagy. Furthermore, phosphoramidon inhibited ER stress and the NLRP3 inflammasome in kidney cells (Figure 7). In a mouse model, phosphoramidon attenuated the progression of CKD by regulating autophagy, the NLRP3 inflammasome and ER stress. Clinically, ETs or ECEs are a potential target for the development of new renoprotective treatments for CKD progression.

## Data Availability

The data presented in this study are available in article or supplementary material.

## Supplementary Materials

The following are available online at https://www.mdpi.com/article/10.3390/biomedicines9040398/s1, Figure S1: Effects of ET-1 or ET-2 exposure on the ER stress in human kidney cells. Figure S2: Effects of ET-1 or ET-2 exposure on the NLRP3 inflammasome in human kidney cells. Figure S3: Effects of phosphoramidon treatment on autophagy in human kidney cells. Figure S4: Effects of phosphoramidon treatment on the thapsigargin-induced ER stress in human kidney cells. Figure S5: Effects of phosphoramidon treatment on the LPS+ATP-induced NLRP3 inflammasom in human kidney cells. Figure S6: Measurement of body weights of C57BL/6 mice in various groups. Figure S7: The ET-1 and ET-2 expression of kidneys after phosphoramidon treatment in the adenine diet-induced CKD model. Figure S8: The high magnification of IHC images. Phosphoramidon regulates ER stress, autophagy and the NLRP3 inflammasome in the adenine diet-induced CKD model. Figure S9: Western blot analysis of ET-1, ET-2, IRE1α, LC3 and NLRP3 in kidney tissue sections in the indicated groups. Table S1: The quantification of immunohistochemical (IHC) and masson staining.
