# Supplementary material for: Therapeutic Effect of Endothelin-Converting Enzyme Inhibitor on Chronic Kidney Disease through the Inhibition of Endoplasmic Reticulum Stress and the NLRP3 Inflammasome
Source: Biomedicines. 2021 Apr 7;9(4):398. doi: 10.3390/biomedicines9040398 (PMC8067871; doi:10.3390/biomedicines9040398)
Supplement: Supplementary file 1 [file biomedicines-09-00398-s001.pdf]

Supplementary Materials: Therapeutic effect of endothelin-converting enzyme inhibitor on chronic kidney disease through the inhibition of endoplasmic reticulum stress and the NLRP3 inflammasome

Yung-Ho Hsu†, Cai-Mei Zheng, Chu-Lin Chou, Yi-Jie Chen, Yu-Hsuan Lee†, Yuh-Feng Lin* and Hui-Wen Chiu*

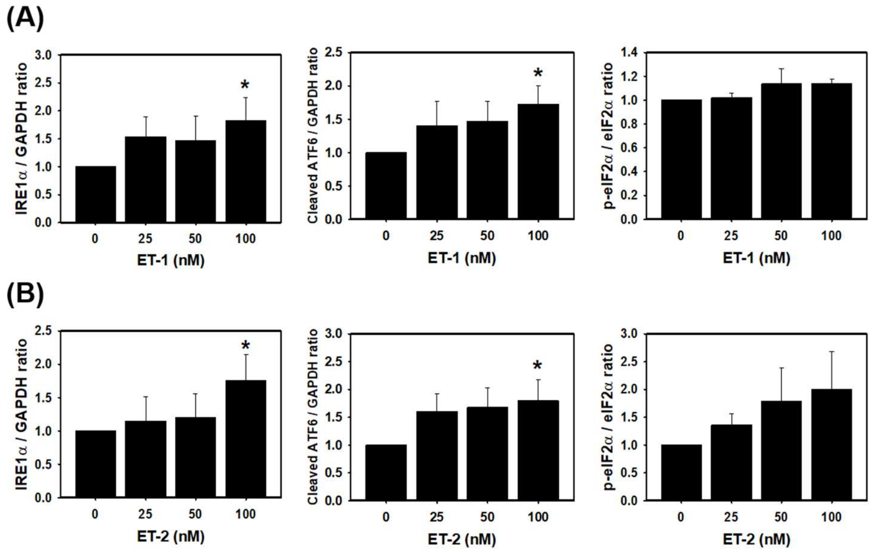

Figure S1. Effects of ET-1 (A) or ET-2 (B) exposure on the ER stress in human kidney cells. The ER stress-related proteins expression of histogram represent the average normalized densitometric values. GAPDH was used as the internal control. Data are presented as the means ± standard deviation of three independent experiments. *P < 0.05 compared with the control.

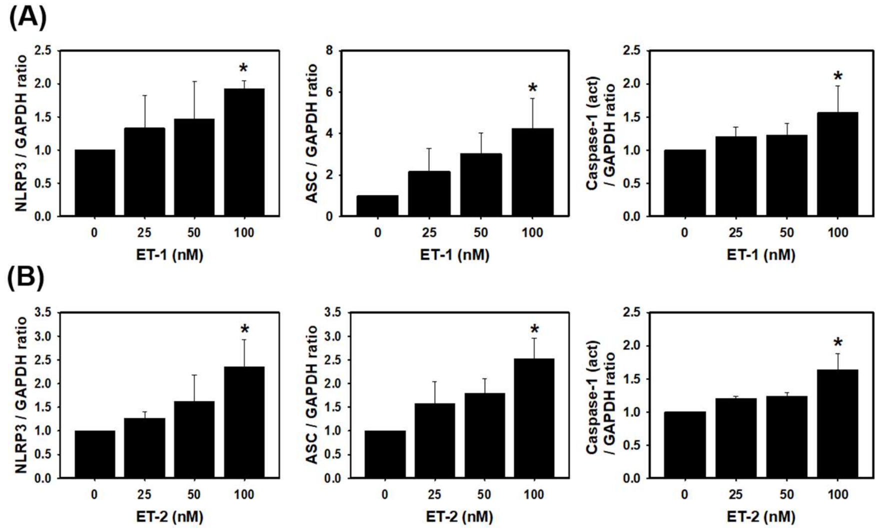

Figure S2. Effects of ET-1 (A) or ET-2 (B) exposure on the NLRP3 inflammasome in human kidney cells. The NLRP3 inflammasome-related proteins expression of histogram represent the average normalized densitometric values. GAPDH was used as the internal control. Data are presented as the means ± standard deviation of three independent experiments. *$P < 0.05$ compared with the control.

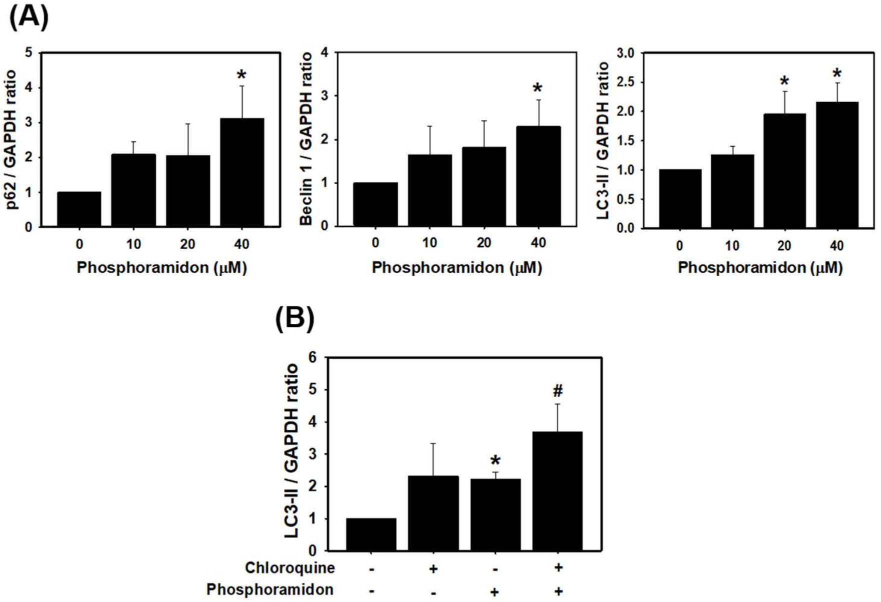

Figure S3. Effects of phosphoramidon treatment on autophagy in human kidney cells. (A) The autophagy-related proteins expression of histogram represent the average normalized densitometric values. *$P < 0.05$ compared with the control. (B) Quantification of LC3-II in HK-2 cells treated with chloroquine and phosphoramidon. The cells were pretreated with chloroquine

(5 μM) for 1 h and then treated with phosphoramidon (20 μM) for 24 h. GAPDH was used as the internal control. Data are presented as the means ± standard deviation of three independent experiments. *P < 0.05 compared with the control. *P < 0.05 compared with the control. # p < 0.05 compared with the phosphoramidon.

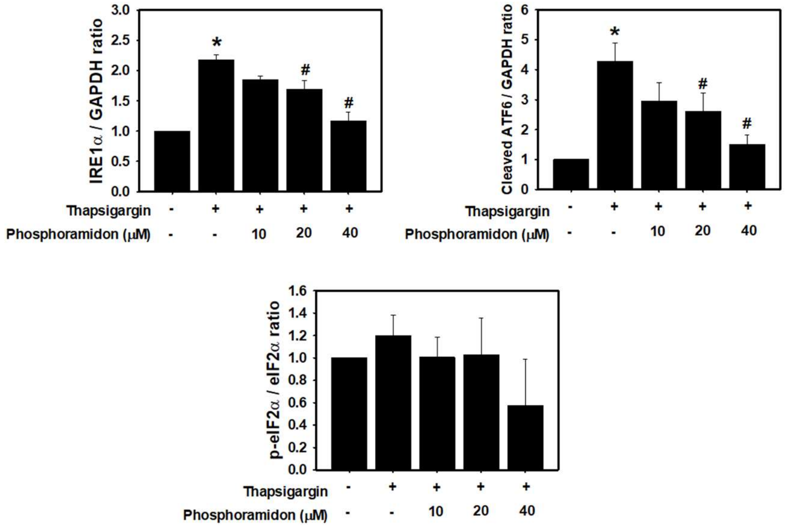

Figure S4. Effects of phosphoramidon treatment on the thapsigargin-induced ER stress in human kidney cells. The ER stress-related proteins expression of histogram represent the average normalized densitometric values. GAPDH was used as the internal control. Data are presented as the means ± standard deviation of three independent experiments. *P < 0.05 compared with the control. # p < 0.05 compared with thapsigargin.

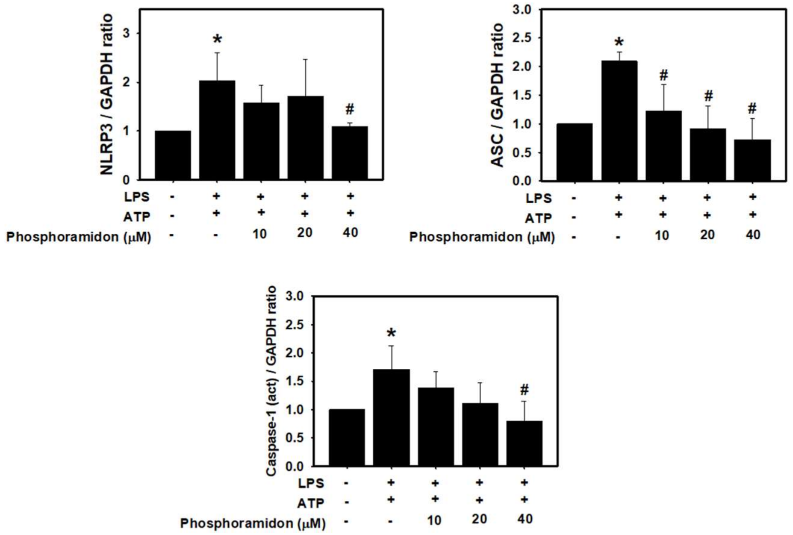

Figure S5. Effects of phosphoramidon treatment on the LPS+ATP-induced NLRP3 inflammasom in human kidney cells. The NLRP3 inflammasom-related proteins expression of histogram represent the average normalized densitometric values. GAPDH was used as the internal control. Data are presented as the means ± standard deviation of three independent experiments. *P < 0.05 compared with the control. # p < 0.05 compared with the LPS+ATP group.

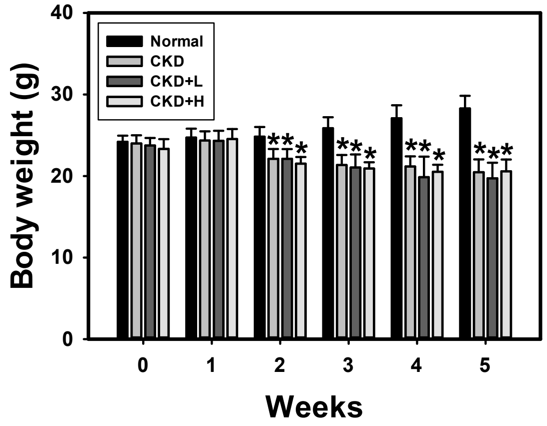

Figure S6. Measurement of body weights of C57BL/6 mice in various groups (5 mice per group). * p < 0.05 compared with the normal group. Data are presented as the means ± standard deviation.

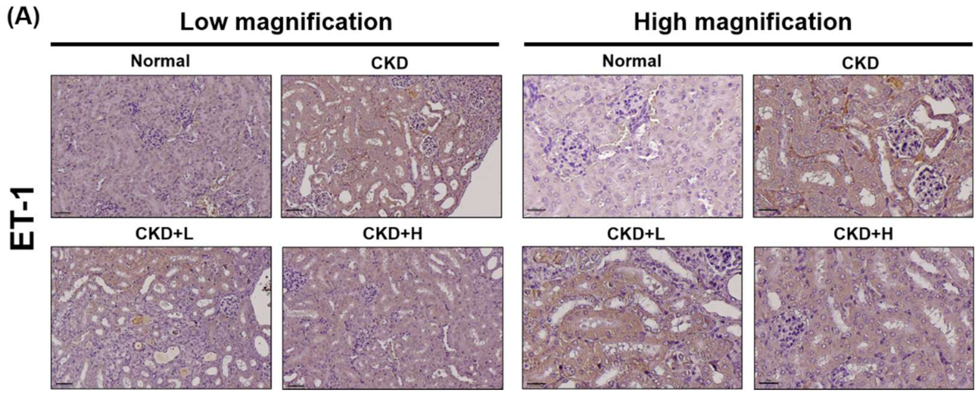

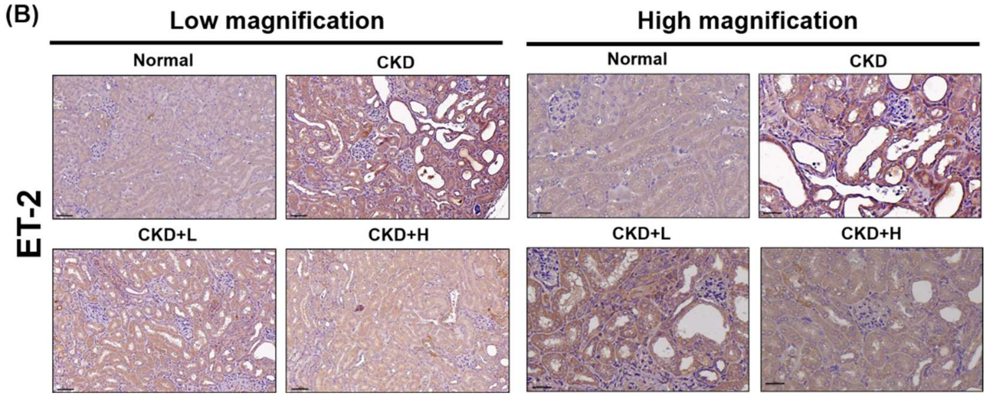

Figure S7. The ET-1 and ET-2 expression of kidneys after phosphoramidon treatment in the adenine diet-induced CKD model. IHC was used to determine the expression levels of ET-1 (A) and ET-2 (B) in kidney tissues. The scale bar of low magnification=60 μm. The scale bar of high magnification=30 μm.

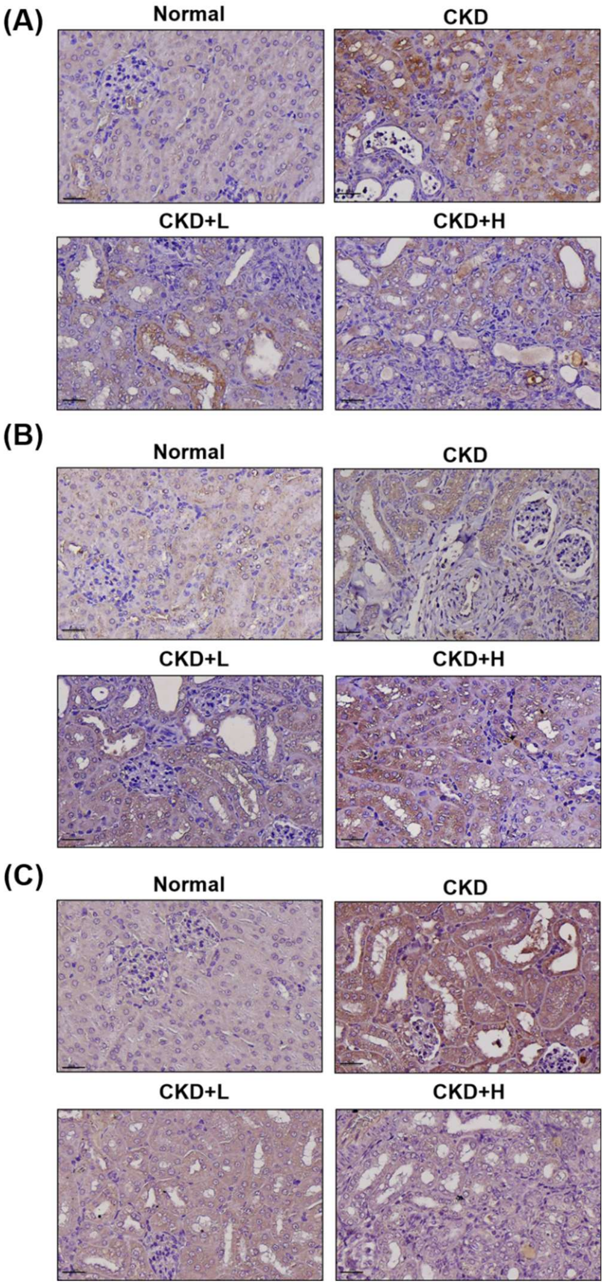

Figure S8. The high magnification of IHC images. Phosphoramidon regulates ER stress, autophagy and the NLRP3 inflammasome in the adenine diet-induced CKD model. The protein expression of IRE1α (A), LC3 (B) and NLRP3 (C) in kidney sections in the indicated groups. Scale bar=30 μm.

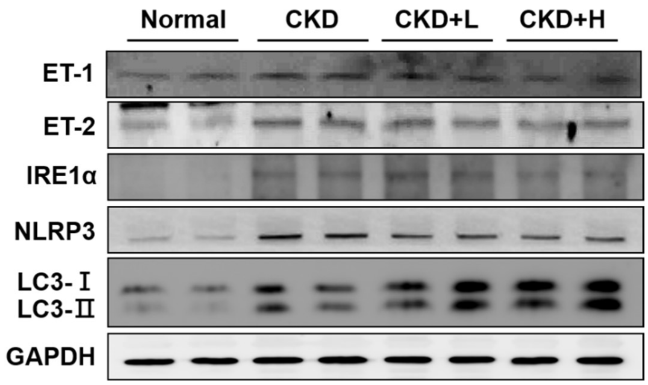

Figure S9. Western blot analysis of ET-1, ET-2, IRE1α, LC3 and NLRP3 in kidney tissue sections in the indicated groups.

Table S1. The quantification of immunohistochemical (IHC) and masson staining.

Item / Group	Normal	CKD	CKD+L	CKD+H
**IRE1α area (%)**	1.11±1.20	7.39±1.37*	6.25±1.72	3.25±1.91#
**LC3 area (%)**	2.10±1.62	5.56±1.66*	7.23±1.47#	8.90±1.35#
**NLRP3 area (%)**	1.91±1.06	26.19±5.87*	14.48±5.86#	10.75±2.97#
**ET-1 area (%)**	3.87±1.39	18.75±5.34*	15.96±5.18	9.10±3.57#
**ET-2 area (%)**	3.50±1.61	35.64±4.09*	21.99±3.19#	10.08±2.33#
**Masson staining positive area (%)**	0.2±0.13	5.8±1.86*	4.21±1.19#	2.02±0.61#

*p<0.05, CKD compared with normal.

#p<0.05, CKD+L or CKD+H compared with CKD.
